# Metagenome Sequences from the Environment of Diseased Otter Clams, Lutraria rhynchaena, from a Farm in Vietnam

**DOI:** 10.1128/MRA.01068-19

**Published:** 2020-01-09

**Authors:** Yuki Kagaya, Ryuhei Minei, Ha T. T. Duong, Binh T. N. Le, Lua T. Dang, Trang T. H. Tran, Hoa T. Nguyen, Kengo Kinoshita, Kei Yura, Atsushi Ogura, Oanh T. P. Kim

**Affiliations:** a Graduate School of Information Sciences, Tohoku University, Sendai, Japan; b Department of Bioscience, Nagahama Institute of Bio-Science and Technology, Nagahama, Shiga, Japan; c Graduate School of Humanities and Science, Ochanomizu University, Tokyo, Japan; d Institute of Genome Research, Vietnam Academy of Science and Technology, Cau Giay, Hanoi, Vietnam; e Research Institute of Aquaculture No. 1, Dinh Bang, Tu Son, Bac Ninh, Vietnam; f Tohoku Medical Megabank Organization, Tohoku University, Sendai, Japan; g School of Advanced Science and Engineering, Waseda University, Tokyo, Japan; Portland State University

## Abstract

Otter clam farming in Vietnam has recently encountered difficulties due to swollen-siphon disease. Here, we report the metagenome sequences of microorganisms extracted from the siphon tissue of infected otter clams. The data comprised bacterial and viral sequences which likely include those derived from the disease-causing agent.

## ANNOUNCEMENT

The otter clam (Lutraria rhynchaena Jonas, 1844), mainly farmed in the central and northern parts of Vietnam, is one of the most economically important bivalve mollusks of the country (1). In 2011, otter clam production reached over 1,200 tons (2), but swollen-siphon disease has raged through the farms, resulting in a huge decline in otter clam aquaculture (3, 4). The identification and extermination of the causative agent of the disease are urgent issues for the recovery of aquaculture, but the cause of the disease is still elusive. Several bacterial species belonging to the genus Vibrio were identified from diseased clams, but a challenge test for clams with the bacteria did not trigger swollen-siphon disease (4). The culprit of the disease is now suspected to be a virus, but evidence of the causative agent has been elusive (4, 5).

We therefore took an alternative approach to finding the causative agent, namely, identifying organisms growing rapidly in and around the diseased clams through genome sequencing. We collected the samples from farms at Cát Bà, Hai Phong (20°43′28.7″N, 107°02′47.3″E) and Vân Đồn, Quảng Ninh (20°57′22.3″N, 107°27′50.1″E) in Vietnam. In each province, we sampled 30 or more individuals at concentrated clam farms. The samples were scrutinized by experts and by electron microscopy to choose the ones with typical swollen-siphon disease. Five infected clams were selected at each site, and the siphon tissues were dissected aseptically, pooled, and homogenized in sterile phosphate-buffered saline (PBS) buffer. The homogenate was centrifuged at 4,000 rpm for 15 min to discard the cell pellet and then filtered through a 0.45-μm syringe filter. DNA was extracted from the filtrate using a PureLink viral RNA/DNA minikit (Invitrogen) and amplified using phi29 DNA polymerase (GenomiPhi; GE Healthcare) and a whole-genome amplification kit (Sigma-Aldrich). A paired-end library was constructed using a TruSeq Nano DNA kit (Illumina), following the manufacturer’s protocols. The DNA library was sequenced with an Illumina HiSeq instrument. A total of 61,607,881 paired-end reads (2 × 151 bp) were obtained. The sequences were mapped to the otter clam genome, which we had already obtained (R. Minei, Y. Kagaya, T. T. H. Tran, H. S. Tran, H. T. T. Duong, B. T. N. Le, L. T. Dang, O. T. P. Kim, K. Kinoshita, A. Ogura, and K. Yura, unpublished data), with BWA v0.7.17 (r1188) (6). Unmapped reads were extracted by SAMtools v1.9 (7). Approximately 3% of the reads (3,393,316 out of 61,607,881 × 2 reads) were not mapped to the otter clam genome sequences. The extracted reads were analyzed by using the Kaiju Web server (last updated 16 May 2017) (8) with default parameters.

The results of the Kaiju analysis, namely, the taxonomic composition of the unmapped reads, are summarized in Table 1. Twenty-four percent of the reads were mapped to known species, mostly to bacterial DNA, a large share of which were *Alphaproteobacteria* and *Staphylococcaceae*, and a few to viral DNA. As mentioned above, we expected that the causative agent was a virus and hence that the results might contain the candidate virus DNA sequences.

**TABLE 1:** Taxonomic assignment of read pairs not mapped to the otter clam genome

Organism^*a*^	No. (%) of read pairs:	
	Unmapped to any sequences	Mapped to sequence in databases
Total	1,293,389 (76)	403,269 (24)
Cellular organisms		399,813 (99)
Archaea/Eukaryota/Fungi		39,140
Bacteria		360,673
*Proteobacteria*		129,102
*Alphaproteobacteria*		60,339
*Terrabacteria*		126,001
*Staphylococcaceae*		74,967
FCB group		73,217
*Flavobacteria*		44,111
Other bacteria		32,353
Viruses		3,254 (1)
Unclassified		984
From environmental samples		767
dsDNA viruses		826
ssDNA viruses		589
Other		124

a FCB, *Fibrobacteres*, *Chlorobi*, and *Bacteroidetes*; dsDNA, double-stranded DNA; ssDNA, single-stranded DNA.

### Data availability.

The raw reads from the metagenomic sequencing are available in the DDBJ Sequence Read Archive under accession number DRA008913.
